# Bipyridine covalent organic framework aerogel for highly selective recovery of palladium in wastewater†

**DOI:** 10.1039/d4sc08674k

**Published:** 2025-03-04

**Authors:** Yang Liu, Weikang Guo, Jiale Liu, Haijuan Tao, Juan Yang, Qin Shuai, Yusuke Yamauchi, Brian Yuliarto, Yusuke Asakura, Lijin Huang

**Affiliations:** a State Key Laboratory of Geomicrobiology and Environmental Changes, Faculty of Materials Science and Chemistry, China University of Geosciences No. 388, Lumo Road, Hongshan District Wuhan 430074 PR China huanglj@cug.edu.cn; b School of Chemistry and Environmental Engineering, Wuhan Institute of Technology LiuFang Campus, No. 206, Guanggu 1st Road, Donghu New & High Technology Development Zone Wuhan 430205 Hubei Province PR China; c Department of Materials Process Engineering, Graduate School of Engineering, Nagoya University Furo-cho, Chikusa-ku Nagoya Aichi 464-8603 Japan asa.y@nagoya-u.jp; d Australian Institute for Bioengineering and Nanotechnology (AIBN), The University of Queensland Brisbane QLD 4072 Australia; e Department of Chemical and Biomolecular Engineering, Yonsei University 50 Yonsei-ro, Seodaemun-gu Seoul 03722 South Korea; f Faculty of Industrial Technology, Institut Teknologi Bandung Bandung 40132 Indonesia

## Abstract

Palladium (Pd), a rare and precious metal, is highly valued due to its non-renewable nature and significant cost. Therefore, recovering palladium from industrial wastewater is of great importance but remains a challenge. Herein, a composite aerogel adsorbent has been developed by linking a bipyridine covalent organic framework, termed TpBpy, with chitosan (CS) through robust covalent bonds. The resulting TpBpy/CS aerogel is employed for the selective separation and recovery of palladium at low concentrations in real wastewater. Experimental results reveal that the maximum adsorption capacity of the TpBpy/CS aerogel for Pd(ii) is 274.4 mg g^−1^ at pH 1. Additionally, even in the presence of other coexisting ions at concentrations 100 times higher than Pd(ii), the adsorption efficiency for Pd(ii) remains above 99%. Mechanistic investigations indicate that the adsorption of Pd(ii) by the TpBpy/CS aerogels primarily occurs through the coordination between pyridine N and Pd(ii), as well as the electrostatic interaction between protonated amino groups and Pd(ii). Moreover, the TpBpy/CS aerogel demonstrates exceptional reusability, maintaining an adsorption efficiency for Pd(ii) above 99% after nine adsorption–desorption cycles. Overall, the TpBpy/CS aerogel is a promising monolithic adsorbent for the efficient recovery of Pd(ii) from acidic industrial wastewater due to its exceptional adsorption capacity and selectivity, demonstrating substantial potential for practical applications.

## Abstract 2

A covalent organic framework chitosan aerogel was prepared and demonstrated high efficiency for selective Pd(ii) recovery from wastewater.

## Graphical abstract

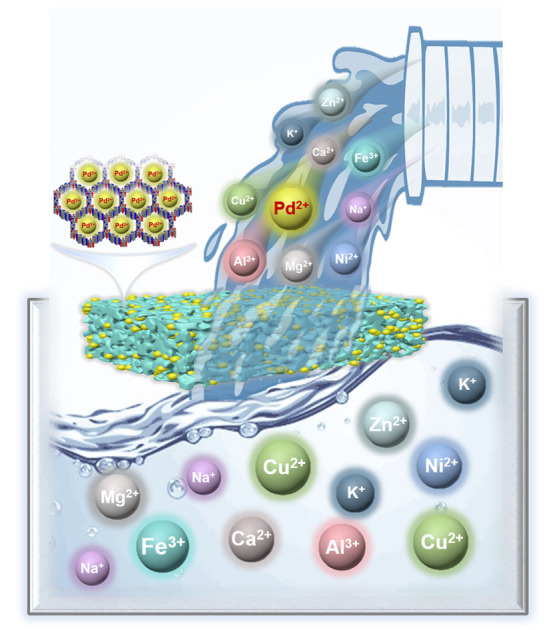

## Introduction

Palladium (Pd) is an exceedingly valuable precious metal, which has been widely used in catalysis,^1^ electronics,^2^ medicine,^3^ and batteries,^4^ due to its unique physical and chemical properties. As a scarce natural resource, Pd exists in the Earth's crust at a remarkably low concentration of only 0.015 parts per million.^5^ With the continuous advancement of various industries, the global demand for Pd has surged, contributing to its consistently high market value. As reported by Johnson Matthey, Pd prices soared to $ 1855 per oz in January 2023.^6^ However, the widespread utilization of Pd inevitably leads to the discharge of significant quantities of industrial wastewater containing Pd into the environment. Although the Pd content in such actual industrial wastewater is low,^7^ if left untreated, it will not only pollute the environment and threaten human health but also result in the wastage of valuable resources.^8^ Consequently, the recovery of Pd from the wastewater holds paramount importance.

A variety of techniques have been investigated for the recovery of Pd(ii) from wastewater, including solvent extraction,^9^ ion exchange,^10^ chemical deposition,^11^ and adsorption.^12^ Among these, the adsorption method stands out due to its straightforward operation, cost-effectiveness, and minimal secondary pollution. To date, a variety of adsorbents have been reported, ranging from porous carbon^13^ and silica functional materials^14^ to biomass adsorbents,^15^ metal–organic frameworks (MOFs),^16^ covalent organic frameworks (COFs),^17^ and others.^18,19^ For instance, the ionic adsorbent COP-1-Cl exhibits rapid adsorption kinetics for Pd(ii), reaching equilibrium in just three minutes (min).^18^ In another case, a covalent isothiocyanate framework, rich in thiourea groups, demonstrated an impressive adsorption capacity of up to 909.1 mg g^−1^ for Pd(ii).^19^ Given that industrial wastewater is typically acidic and contains a complex mixture of ions, adsorption materials must meet stringent requirements for chemical stability and selectivity. Consequently, it is imperative to develop a low-cost, chemically stable and highly selective adsorbent.

COFs are porous organic polymers that have been widely studied due to their substantial specific surface area, ease of functionalization, and exceptional chemical stability.^20,21^ Given that 2,2′-bipyridine and its derivatives are neutral ligands that can be readily functionalized, several bipyridine-based materials, such as bipyridine functionalized macroporous silica and bipyridine-based porous organic polymers have been rationally designed and used for the adsorption of various metal ions.^22–26^ Additionally, taking advantage of the strong affinity between N and Pd(ii), several COFs containing functional groups like amino groups and pyridine, have been designed and synthesized for the selective adsorption of Pd(ii).^17,23,27^ For instance, a three-component COF (ECUT-COF-34) was recently constructed using a substituent method.^28^ This material exhibits high selectivity for Pd(ii) even at an acidic concentration of 3 mol L^−1^. However, a notable limitation of most current COFs is their powdery form, which complicates the separation from solution and limits their practical utility in precious metal recovery.^29^ Furthermore, the agglomeration of COF powders can occur due to the inter-particle forces. This would reduce the accessible active binding sites and surface area of the COF, which in turns compromises the adsorption capacity and mass transfer efficiency.^30–32^ Additionally, the high production cost poses a barrier to their industrial application. Consequently, there is growing interest in integrating powdered COFs with other low-cost materials to create monolithic composites that combine the advantages of both. This approach addresses the challenges of recovering powdered COF, the agglomerate issue, and cost concerns.^33^ To date, a variety of monolithic COF materials have been investigated, including COF/chitosan (CS) aerogels,^34^ COF/graphene oxide aerogels,^35^ COF/melamine sponges,^36^ and so on. Among them, COF/CS aerogels have garnered significant interest due to their distinct characteristics. CS, derived from the partial *N*-deacetylation of chitin, is a naturally occurring polysaccharide, which is rich in amino and hydroxyl groups.^37^ It offers several advantages, such as wide availability, low cost, and non-toxicity.^38^ The abundant amino groups can be used to link with other organic groups, facilitating the creation of strong three-dimensional network structures and enhancing the performance of composites.^39^ Recently, a CS freeze-gel sorbent (VP-AMPS-CS5) was used to adsorb Pd(ii) in electronic waste, demonstrating a maximum adsorption capacity of 184.93 mg g^−1^ at pH = 3.^40^ Nevertheless, the potential application of COF/CS aerogel for Pd(ii) recovery remains underexplored and warrants further investigation.

**Scheme 1:**
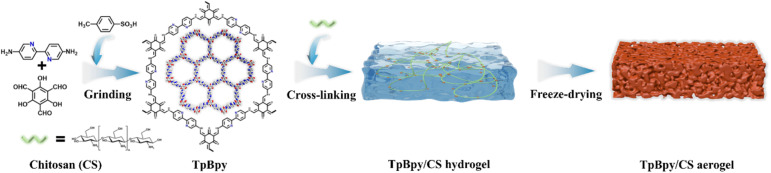
Schematic diagram of the TpBpy/CS aerogel synthesis.

Therefore, in this study, a series of COF/CS aerogels are prepared by covalently linking pyridine COF with CS, using 1,3,5-triformylphloroglucinol (Tp) as a cross-linking agent (Scheme 1). The resulting COF/CS aerogel was subsequently employed to selectively recover Pd(ii) from actual wastewater. Batch adsorption experiments were conducted to investigate the adsorption kinetics and isotherms of Pd(ii), as well as the effect of pH on adsorption performance. Simultaneously, the adsorption selectivity of the materials toward Pd(ii) in the actual wastewater was evaluated. Additionally, the reusability of the material towards Pd(ii) was verified through adsorption and desorption experiments.

## Results and discussion

### Synthesis and characterization

**Fig. 1:**
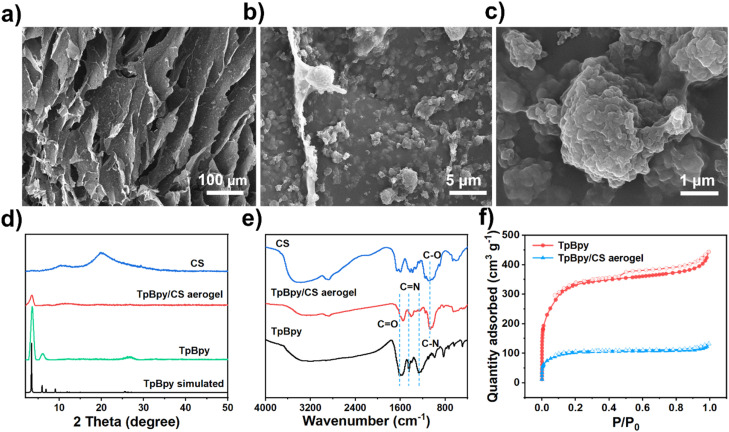
(a–c) SEM micrographs of TpBpy/CS aerogel; (d) XRD patterns and (e) FT-IR spectra of CS, TpBpy, and TpBpy/CS aerogel; (f) N_2_ adsorption–desorption isotherms of TpBpy/CS aerogel and TpBpy.

The synthesis of TpBD, TpBpy, Tp/CS aerogel, and TpBpy/CS aerogel is described in detail in the Methods section. As shown in Fig. S1,† the TpBpy/CS aerogel can stand upright on green bristlegrass without bending its fluff, attributed to its low density (0.026 g cm^−3^). Additionally, the uniform color across the TpBpy/CS aerogel indicates that TpBpy powders are evenly distributed within the CS network. Meanwhile, as shown in Fig. 1a–c, scanning electron microscope (SEM) micrographs illustrate various regions of the TpBpy/CS aerogel, further confirming the uniform distribution of TpBpy powders within the polymeric networks of CS. These observations suggest that the agglomeration of COF powders is minimized. Furthermore, these network structures create numerous pores, which enhance the exposure of additional adsorption sites and improve the adsorption performance of TpBpy/CS aerogel. In addition, the hierarchical pore structure also facilitates substances diffusion, further improving the adsorption efficiency. To verify the successful preparation of the TpBpy/CS aerogel composite, the material was characterized using X-ray diffraction (XRD), Fourier Transform Infrared (FT-IR) spectroscopy, and N_2_ adsorption–desorption measurements. The XRD patterns of the TpBpy/CS aerogel, TpBpy powder, and CS are presented in Fig. 1d. Both the TpBpy/CS aerogel and the TpBpy powder exhibit a characteristic pattern at 3.6°, corresponding to the (100) plane. The sharp pattern originating from the (100) plane is consistent with the simulated pattern of TpBpy and the previously reported results, indicating the well-formed crystal structure of TpBpy.^41^ Additionally, a broad pattern belonging to the plane (001) at 26.1° is observed, attributed to the π–π stacking of TpBpy lamellae.^42^ The intensity of the diffraction pattern at 3.6° in the TpBpy/CS aerogel is lower than that of TpBpy powder, due to the presence of CS molecules. In the FT-IR spectra shown in Fig. 1e, bands at 1656 cm^−1^ and 1262 cm^−1^ are observed, corresponding to the stretching vibration of C=O and C–N bonds, respectively.^43^ The band at 1422 cm^−1^ is attributed to the C=N stretching frequency in the pyridine structure.^44^ The band at 1066 cm^−1^ indicates the C–O bond stretching vibration in CS.^45^ These results collectively demonstrate the successful synthesis of the TpBpy/CS aerogel.

The porosity and specific surface area of powdery TpBpy and TpBpy/CS aerogel were investigated by the N_2_ adsorption and desorption isothermal curves at 77 K (Fig. 1f). The N_2_ adsorption and desorption isotherms of TpBpy are consistent with type I. When the relative pressure P/P_0_ is in the range of 0–0.1, a significant N_2_ adsorption is observed, indicating the presence of numerous microporous structures.^46^ In addition, a hysteresis loop is observed, which may potentially be caused by the accumulation of COF particles.^47,48^ The pore size distributions of both TpBpy/CS aerogel and powdery TpBpy were calculated using the nonlinear density functional theory (NLDFT) method, revealing a range of pore sizes spanning from microporous to mesoporous structures (Fig. S2†). The Brunauer–Emmett–Teller (BET) specific surface area and pore volume of the TpBpy/CS aerogel are 332.7 m^2^ g^−1^ and 0.191 cm^3^ g^−1^, respectively. In comparison, the BET-specific surface area and pore volume of TpBpy are 1064.3 m^2^ g^−1^ and 0.687 cm^3^ g^−1^, respectively. The decrease in the BET-specific surface area and pore volume for TpBpy/CS aerogel is attributed to the pore blocking by CS polymers.

To demonstrate the potential of TpBpy/CS aerogel for practical applications, it is essential to evaluate its thermal and chemical stability. As illustrated in Fig. S3,† the Tp/CS aerogel exhibits a weight loss of approximately 30% up to 300 °C, primarily due to the adsorbed water and the chain polymerization of CS.^49^ Beyond 300 °C, a significant decrease in weight is observed, which is consistent with the results of previous studies.^50^ Additionally, TpBpy demonstrates excellent thermal stability up to 416 °C. When combined with CS to form an aerogel, the resulting TpBpy/CS aerogel exhibits excellent thermal stability up to 330 °C. At approximately 200 °C, a weight loss of 11.5% is observed for the TpBpy/CS aerogel, attributed to the volatilization of the physically adsorbed water or residual solvent. Beyond 400 °C, a gradual decrease in weight is observed, indicating the decomposition of the aerogel framework.^51^ These findings demonstrate the good thermal stability of TpBpy/CS aerogel. To evaluate the chemical stability of the TpBpy/CS aerogel, TpBpy/CS aerogels were soaked in 0.1, 1.0 and 5.0 mol L^−1^ HCl and HNO_3_ solution for 24 hours (h). The soaked TpBpy/CS aerogels were analyzed by XRD, FT-IR and N_2_ adsorption–desorption isotherms. As shown in Fig. S4a and b,† after soaking in HCl and HNO_3_ solution at different concentrations for 24 h, no significant changes are observed in the FT-IR spectra of the TpBpy/CS aerogel. However, some changes may be present in some of the PXRD patterns. Additionally, as illustrated in Fig. S4c,† the BET specific surface area of TpBpy/CS aerogels decrease from 332.7 m^2^ g^−1^ to 206.3 m^2^ g^−1^ and 57.8 m^2^ g^−1^ after soaking in 0.1 and 1.0 mol L^−1^ HNO_3_ solution for 24 h, respectively. This decrease may be attributed to partial decomposition of COF within the TpBpy/CS aerogel.

### Adsorption investigations

To demonstrate that the integration of COF and CS into aerogel enhances the adsorption performance of the material, the adsorption capacities of Tp, TpBD, CS, Tp/CS aerogel, TpBpy, and TpBpy/CS aerogel for Pd(ii) were compared at pH = 1. As shown in Fig. S5,† the adsorption capacities of Tp, TpBD, TpBpy, CS, Tp/CS aerogel, and TpBpy/CS aerogel for Pd(ii) are 26.5 mg g^−1^, 110.9 mg g^−1^, 243.3 mg g^−1^, 56.1 mg g^−1^, 137.3 mg g^−1^, and 274.4 mg g^−1^, respectively. Notably, the adsorption capacity of CS is significantly lower than that of TpBpy. Considering the amount of COF in the resultant TpBpy/CS aerogel, the adsorption capacity corresponding to COF within the aerogel is calculated to be 411.6 mg g^−1^, which is substantially higher than that of parent COF-TpBpy (243.3 mg g^−1^). This result suggests that the adsorption performance is enhanced due to the reduced agglomeration of COF powders, as also evidenced by SEM characterization (Fig. 1a–c). Simultaneously, TpBD, which lacks pyridine moieties, exhibits inferior adsorption capacity for Pd(ii), indicating that pyridine plays an essential role in the adsorption process.

To determine the optimal mass loading of COF in the aerogel, a series of TpBpy/CS-*X* aerogels (*X* = 0.2, 0.4, 0.5, 0.6, and 0.8) were synthesized, and their adsorption capacities for Pd(ii) were compared (Table S1†). As shown in Fig. S6a,† when the COF content in TpBpy/CS aerogel is below 50%, some COF particles are coated by CS, blocking the active sites and preventing their full utilization. However, when the COF content in TpBpy/CS aerogel exceeds 50%, the COF particles tend to agglomerate, resulting in incomplete exposure of the adsorption sites. When the COF content of the TpBpy/CS aerogel is 50%, the COF powders are uniformly distributed within the CS network, resulting in a higher adsorption capacity. To further confirm that the construction of COF/CS aerogel can improve the adsorption capacity of COF for Pd(ii), the adsorption capacities of COF for Pd(ii) in TpBpy/CS aerogel with varying COF content were evaluated. As shown in Fig. S6b,† the adsorption capacities corresponding to COF significantly increase, due to the uniform distribution of COF powders within the CS network, which mitigates the agglomeration of COF particles. However, when the COF content exceeds 50%, the adsorption capacity corresponding to COF gradually decreases, likely due to the agglomeration of COF powders hindering the full exposure of adsorption sites. Considering that the preparation cost of COF is much higher than that of CS, it is crucial to balance the preparation cost and the adsorption capacity of COF/CS aerogel. Thus, a COF/CS aerogel with 50% COF content was selected for subsequent experiments, providing an optimal balance between performance and cost-effectiveness.

**Fig. 2:**
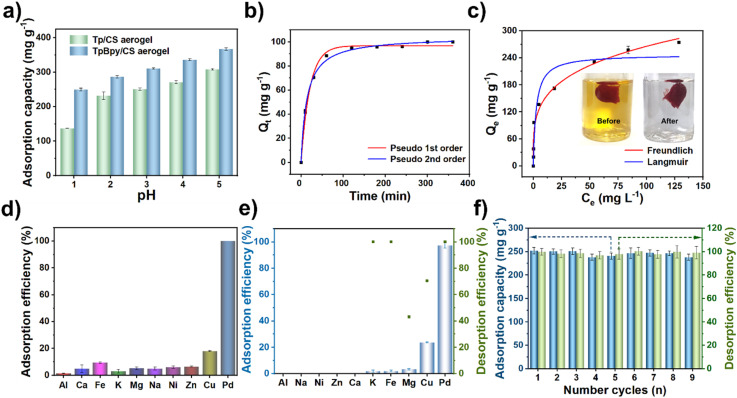
(a) Effect of pH on adsorption of Pd(ii) (conditions: adsorbent dosage: 5 mg; *V* = 10.0 mL; *C*_Pd_ = 250 mg L^−1^; temperature: 25 °C; time: 24 h); (b) the pseudo 1st order kinetics and pseudo 2nd order kinetics models of Pd(ii) adsorption by TpBpy/CS aerogel; (c) the Freundlich and Langmuir model of Pd(ii) adsorption by TpBpy/CS aerogel (the inserted photograph shows the color change of the solution before and after the adsorption of Pd(ii) by TpBpy/CS aerogel); (d) adsorption selectivity (conditions: adsorbent dosage: 5 mg; *V* = 10.0 mL; *C*_Pd_ = 10 mg L^−1^; *C*_other_ = 1000 mg L^−1^; temperature: 25 °C; pH = 1; time: 24 h); (e) adsorption and desorption efficiency of TpBpy/CS aerogel in actual metallurgical wastewater; (f) reusability of the TpBpy/CS aerogel.

The pH of the solution plays a crucial role in determining the charge state of the adsorbent material and the speciation of Pd(ii), both of which significantly influence the material's ability to adsorb and separate Pd(ii).^52^ In the pH range of 1–5, Pd(ii) predominantly forms chlorides by combining with Cl^−^ ions. As the pH increases, the concentration of OH^−^ ions rises, promoting the formation of Pd(ii) hydroxide precipitates.^27,53^ Given that industrial wastewater typically exhibits acidic conditions, the pH range of 1–5 was selected to investigate the adsorption capacity of TpBpy, TpBpy/CS aerogel and Tp/CS aerogel for Pd(ii). As depicted in Fig. 2a and S7,† the adsorption capacities of TpBpy/CS aerogel are higher than those of TpBpy across the pH range of 1–5. Furthermore, the adsorption capacities of both TpBpy/CS aerogel and Tp/CS aerogel for Pd(ii) gradually increase as the pH increases. Notably, under acidic conditions, the amino groups in the adsorbent become protonated. This protonation leads to a positively charged material, which facilitates electrostatic adsorption of PdCl_4_^2−^ ions.^54^ However, two effects can reduce the adsorption capacity for Pd(ii) species. On one hand, the high concentration of H^+^ in the solution combines with PdCl_4_^2−^, preventing the Pd(ii) species from effectively binding with the adsorbent. On the other hand, Pd(ii) predominantly exists in the form of PdCl_4_^2−^ under such acidic conditions, and a large amount of Cl^−^ ions present in the solution would competitively combine with the active binding sites, further decreasing the adsorption capacity.^8,55,56^

As shown in Fig. S8,† the pH_PZC_ value (pH value of the point of zero charge) of TpBpy/CS aerogel is determined to be 4.86, indicating that the aerogel becomes positively charged at pH values below 4.86, and negatively charged at pH values above 4.86.^12^ Considering that Pd(ii) remains electronegative in acidic solutions,^57^ the electrostatic interaction between TpBpy/CS aerogel and Pd(ii) species enhances adsorption under acidic conditions. Furthermore, at pH = 1, the adsorption capacity of TpBpy/CS aerogel significantly exceeds that of Tp/CS aerogel, highlighting the superior adsorption capacity of COF with the bipyridine structure for Pd(ii) at low pH. Given the prevalent acidic nature of actual wastewater, pH 1 was selected for subsequent experiments.

The time required to reach adsorption equilibrium is a vital parameter for evaluating the performance of adsorbents. Therefore, the adsorption kinetics was investigated. As depicted in Fig. 2b, the adsorption process primarily occurs within the first 60 min. The adsorption equilibrium time for TpBpy/CS aerogel is slightly longer than that of powdered TpBpy.^58^ This disparity can be attributed to the better dispersion of powder particles in solution, resulting in faster equilibration. However, as a monolithic adsorbent, TpBpy/CS aerogel offers greater ease for separation and recovery. During the initial stage of the adsorption process, the adsorption rate is fast due to the presence of numerous active sites on the TpBpy/CS aerogel surface available for binding Pd(ii). This is due to the uniform distribution of TpBpy particles within the CS polymeric networks, ensuring the full exposure of the adsorption sites. To further evaluate its dynamic behavior, both the pseudo 1st order and pseudo 2nd order kinetic models were employed for data analysis and the results are summarized in Table S2.† The *R*^2^ value for the pseudo 2nd order kinetic model (0.996) is greater than that for the pseudo 1st order kinetic model (0.991), indicating that the surface of TpBpy/CS aerogel contains abundant adsorption active sites.^59^

To determine the maximum adsorption capacity of the material for Pd(ii), an adsorption isotherm experiment was conducted using initial concentrations ranging from 10 to 250 mg L^−1^. According to Fig. 2c, the adsorption capacity of the aerogel for Pd(ii) initially increases with the increase of concentration, and then gradually reaches the adsorption equilibrium. The maximum adsorption capacity of TpBpy/CS aerogel for Pd(ii) is determined to be 274.4 mg g^−1^. Nonlinear fitting of the isotherm data was performed using both the Langmuir and the Freundlich models, with the relevant parameters presented in Table S3.† The results suggest that the adsorption behavior of TpBpy/CS aerogel towards Pd(ii) aligns with the Freundlich model's characteristics. This alignment is attributed to the porosity and heterogeneity of the TpBpy/CS aerogel, which is caused by the random cooling processes during its preparation.^40,60,61^ This observation is consistent with the SEM micrographs shown in Fig. 1a–c. A comparison of the adsorption capacities of other adsorbents for Pd(ii) reported in the literature is summarized in Table S4.† Compared with other adsorbents such as Tp-DG_Cl_,^62^ and ECUT-COF-34,^28^ TpBpy/CS aerogel maintains high adsorption capacity and fast adsorption kinetics for Pd(ii) at low pH.

Since interfering ions are commonly present in actual industrial wastewater, the selectivity of the adsorbent must be evaluated. As shown in Fig. 2d, the TpBPy/CS aerogel exhibits nearly complete adsorption of Pd(ii) even in the presence of coexisting ions at concentration 100 times higher, highlighting its excellent selectivity for Pd(ii) at low concentrations. The high selectivity can be attributed to the presence of a large number of bipyridine structures within the aerogel, where the N atom displays a strong affinity for Pd(ii). This mechanism will be explored in the mechanistic analysis section. Meanwhile, the selectivity of CS and TpBpy for Pd(ii) under the same conditions was evaluated (Fig. S9†). The experimental results reveal that CS alone adsorbs only 44.6% of Pd(ii) in the presence of interfering ions, whereas TpBpy captures almost 100% of Pd(ii). This result further reinforces the significance of incorporating TpBpy COF with pyridine groups to enhance the selectivity of the resulting COF/CS aerogel towards Pd(ii).

To further verify the practical application potential of TpBpy/CS aerogel for Pd(ii) recovery, the adsorption capacities of the TpBpy/CS aerogel for Pd(ii) were evaluated under varying solution volumes. As shown in Fig. S10a,† the adsorption capacities of TpBpy/CS aerogel for Pd(ii) gradually increase with increasing the solution volume. This suggests that the TpBpy/CS aerogel maintains an excellent adsorption capacity for low-concentration Pd(ii) (10 mg L^−1^) in large solution volumes, meeting the requirements for practical applications, especially in the treatment of large-scale wastewater. As illustrated in Table S5,† the adsorption efficiency of TpBpy/CS aerogel for Pd(ii) remains above average levels even at these low concentrations. Furthermore, as shown in Fig. S10b,† although the distribution coefficient (*K*_d_) value of Pd(ii) decreases as the volume increases, it remains above 1.0 × 10^4^, which is significantly higher than other ions' *K*_d_ values (<1.0 × 10^3^). For further analysis, the separation factors (SF_Pd/M_) were calculated and presented in Table S6.† The higher SF_Pd/M_ values indicate that the TpBpy/CS aerogel demonstrates enhanced Pd selectivity and greater anti-interference ability in complex environments. These results demonstrate that the TpBpy/CS aerogel maintains high selectivity even in large solution volumes. Additionally, the monolithic nature of the TpBpy/CS aerogel facilitates its recycling and reuse. These findings highlight the great potential of TpBpy/CS aerogel for applications in the separation and recovery of Pd(ii) from real-world wastewater.

To evaluate its practicality, adsorption experiments were conducted using metallurgical wastewater from the Jinbao Mountain palladium–platinum deposit in Yunnan Province, China. The pH of the wastewater was adjusted to 1, and no precipitation was observed (Table S7†). As shown in Fig. 2e, the material retains >95% Pd(ii) selectivity in complex matrices. The *K*_d_ of TpBpy/CS aerogel for Pd(ii) was calculated to be 36 892, revealing its excellent selectivity for Pd(ii) capture (Table S8†). Although some other metal ions were also adsorbed, their concentration decreased significantly (Table S7†), enhancing Pd(ii) purification through multiple cycles.

The recycling of adsorbents is crucial for promoting environmental sustainability. Experimental results demonstrate that a mixed solution containing 0.5 mol L^−1^ HNO_3_ and 0.5 mol L^−1^ thiourea can effectively elute the Pd(ii) adsorbed on the material (Fig. 2f). This highlights the ease of reclaiming Pd(ii) and the renewability of the adsorbent. For the practical application, the costs of the chemicals used in producing the TpBpy/CS aerogel are listed in Table S9.† Based on a rough estimation, the cost of TpBpy is about 103.5 $ per g. On the contrary, the rough cost of CS is significantly lower, only 0.178 $ per g. Combining TpBpy with CS to form an aerogel reduces the agglomeration of COF particles. This not only improves the adsorption capacity of COF but also significantly reduces the preparation cost. Importantly, the selectivity of the TpBpy/CS aerogel for Pd(ii) remains unchanged (Fig. 2d and e). With these advantageous characteristics, TpBpy/CS aerogel holds promising prospects for the recovery of Pd(ii) from real-world wastewater, further contributing to environmental sustainability.

### Mechanism analysis

**Fig. 3:**
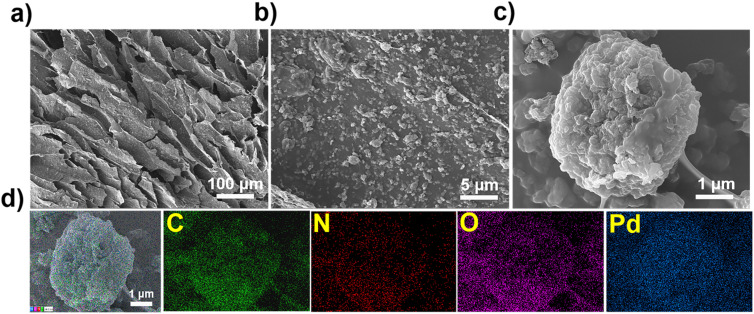
(a–c) Pd(ii)-loaded TpBpy/CS aerogel; (d) SEM micrographs and the corresponding EDS mapping images of Pd(ii)-loaded TpBpy/CS aerogel.

**Fig. 4:**
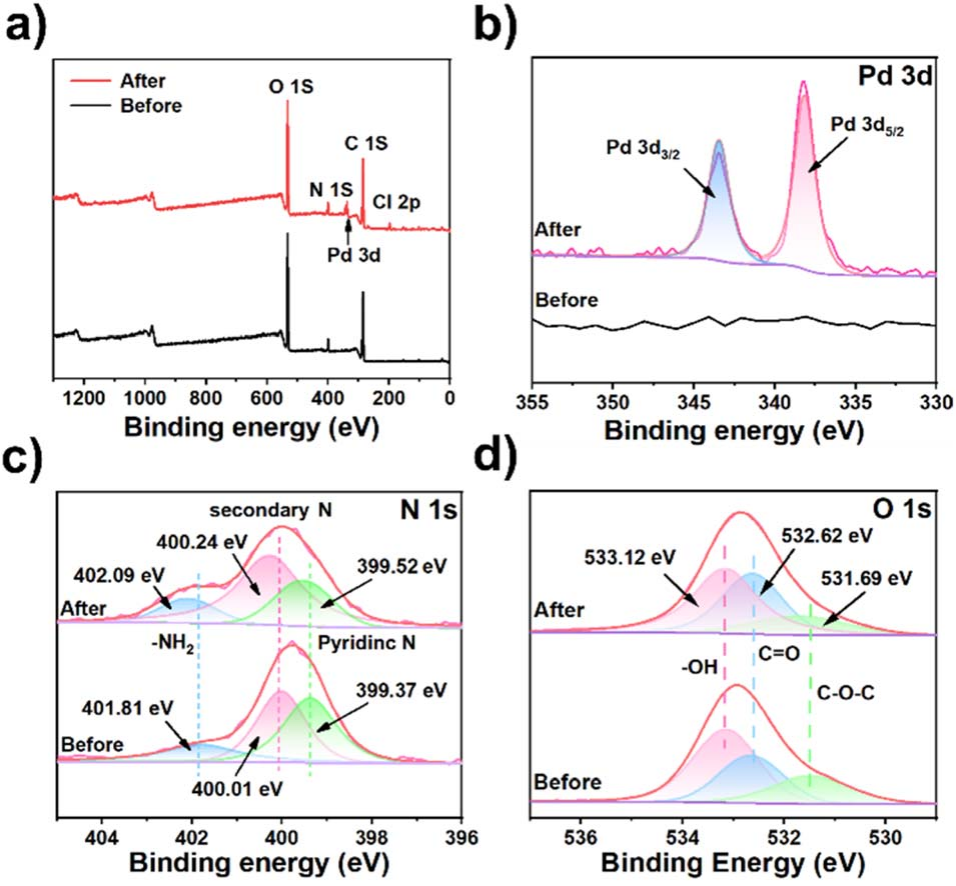
XPS analysis of TpBpy/CS aerogel before and after Pd(ii) adsorption: (a) full-scan spectra, and high-resolution spectra of (b) Pd 3d, (c) N 1s, (d) O 1s.

The adsorption mechanism of Pd(ii) by TpBpy/CS aerogel was elucidated through comprehensive analysis using X-ray photoelectron spectroscopy (XPS), SEM, and FT-IR characterization techniques. SEM micrographs (Fig. 3a–c) of the Pd(ii)-loaded TpBpy/CS aerogel show that the material's morphology remains similar to its original form, indicating robust material stability. The energy-dispersive spectroscopy (EDS) elemental mapping images (Fig. 3d) further confirm the successful adsorption of Pd(ii) onto the aerogel surface. Comparison of FT-IR spectra before and after Pd(ii) adsorption reveals stretching vibrations of C–N bonds (Fig. S11†), suggesting an interaction between N and Pd(ii).^63^ In addition, as depicted in Fig. 4a, XPS spectra after adsorption confirm the successful adsorption of Pd(ii) onto the TpBpy/CS aerogel. Two peaks corresponding to Pd 3d_3/2_ and Pd 3d_5/2_ at the binding energy of 343.42 eV and 338.12 eV, respectively (Fig. 4b), indicate the presence of Pd(ii).^64^ Additionally, analysis of the high-resolution XPS spectrum of N 1s (Fig. 4c) reveals shifts in binding energies post-adsorption, attributed to coordination and electrostatic interactions between N and Pd(ii).^65,66^ Under low pH conditions, part of the pyridine N becomes protonated and positively charged, allowing it to combine with the negatively charged PdCl_4_^2−^ through electrostatic interaction. Additionally, pyridine N as an electron donor provides electrons to the electron-deficient PdCl_4_^2−^, facilitating coordination bonding between pyridine N and Pd(ii). Notably, after Pd(ii) loading, the binding energy of pyridine N shifts from 399.37 eV to 399.52 eV, indicating both coordination and electrostatic interaction between pyridine N and Pd(ii).^58^ Secondary N on TpBpy/CS aerogel is also involved in the adsorption process, and its binding energy is shifted from 400.01 eV to 400.24 eV. The binding energy of –NH_2_ shifts from 401.81 eV to 402.09 eV, which can be attributed to the electrostatic interaction between protonated amino and Pd(ii).^67^ As shown in Fig. 4d, three distinct peaks appear in the high-resolution spectra of O 1s, corresponding to C–O–C, C=O, and –OH, respectively.^49,68^ After Pd loading, the binding energy of C–O–C shifts slightly to a higher value, from 531.45 eV to 531.68 eV, indicating a transfer of electron density from oxygen atoms to Pd(ii).^49^ These findings suggest that the O atoms also participate in the adsorption process of Pd(ii). In conclusion, the main adsorption mechanisms of TpBpy/CS aerogel involve electrostatic interaction and coordination between N, O and Pd(ii).

## Conclusions

In this work, a monolithic COF/CS aerogel material is prepared to address the recycling challenge associated with powder materials. The incorporation of pyridine groups in TpBpy/CS aerogel enables efficient and selective adsorption of Pd(ii) even under acidic conditions. Moreover, the monolithic nature of TpBpy/CS aerogel allows for easy reuse, offering distinct advantages over its powdered counterparts. Furthermore, the material exhibits high selectivity for Pd(ii) adsorption in complex industrial wastewater matrices. Consequently, this research offers a novel approach for recovering various precious metals from low-concentration wastewater, suggesting broad implications for future studies in the field.

## Methods

### Reagents and characterizations

CS, acetic acid, *N*,*N*-dimethylacetamide (DMAc), chloroauric acid, nitric acid, potassium nitrate, zinc nitrate, iron nitrate, nickel nitrate hexahydrate, acetone, calcium nitrate, magnesium sulfate, aluminum nitrate nine hydrate, were obtained from Sinopharm Chemistry Reagent Co. Ltd (China). Copper nitrate was purchased from Tianjin Baishi Chemical Co. Ltd. Benzidine (BD) and *p*-toluenesulfonic acid (PTSA) were purchased from Aladdin (Shanghai, China). The organic monomers including 1,3,5-triformylphloroglucinol (Tp) and 2,2′-bipyridine-5,5′-diamine (Bpy) were acquired from Yanshen Technology (Jilin, China). Palladium chloride was purchased from Anergy Chemical (Shanghai, China). All chemicals were of analytical grade and used directly without purification.

XRD data were obtained using the Ultima IV instrument with Cu K_α_ radiation at a scanning speed of 2° min^−1^ in the range of 2–60°. A NICOLET 5700 FT-IR spectrometer (Thermo Fisher Scientific, the USA) was used to scan the samples in the wavenumber range of 4000–400 cm^−1^ to obtain the FT-IR spectra. The morphology and porous structure of the samples were identified using a field emission SEM on ZEISS Gemini 300 at a working voltage of 10 kV. Nitrogen adsorption–desorption experiments were performed using Autosorb-iQ (Quantachrome Instruments, the USA) at 77 K after the samples were degassed at 120 °C under vacuum for 8 h. The surface area was estimated by the BET method. The NLDFT method was employed to estimate the pore size distribution. Thermogravimetric analysis was performed on a thermal analyzer (STA 449 F5, Netzsch Germany) from room temperature to 800 °C at a heating rate of 10 °C min^−1^. The concentration of metal ions was tested using EXPEC 6000 Inductively Coupled Plasma-Optical Emission Spectrometer (ICP-OES) (Hangzhou PuYu Technology Development Co. Ltd China). The XPS spectra were collected on a Thermo ESCALAB 250 instrument using Al-Kα as the exciting radiation, and binding energy calibration was based on C 1s at 284.8 eV.

### Synthesis of materials

The bipyridine COF TpBpy was prepared *via* a slightly modified method developed by Banerjee *et al.*^31^ Specifically, 2.5 mmol PTSA (430.5 mg) and 0.45 mmol Bpy (83.7 mg) were added to a mortar, and thoroughly ground for 5 min until they were well mixed. Then, 0.3 mmol Tp (63.0 mg) was added to the mortar and ground for another 20 min. After that, 100 μL of water was added and the mixture was ground into a dough-like consistency. The resulting mixture was transferred to a headspace vial and heated at 170 °C for 5 min. The product was then washed successively with hot water, DMAc, and acetone, and dried under vacuum at 60 °C for 12 h to obtain the red powder TpBpy. A similar procedure was used to prepare the COF without pyridine moieties, TpBD, through the condensation of Tp and benzidine (BD). The TpBpy/CS aerogel was synthesized using a procedure based on the methodology previously reported by our research group.^69^ The specific process involves the following steps: initially, 10 mg of CS was dissolved in an acetic acid solution (600 μL, 0.08 mol L^−1^) to create a homogeneous and transparent aqueous solution. Subsequently, 10 mg of TpBpy powder was added to the CS solution and evenly dispersed after 30 seconds of vortexing and sonication. Following this, an aqueous solution containing Tp was prepared by dispersing 2 mg of Tp into 200 μL ultrapure water, which was then added to the mixture. The resultant mixture was allowed to stand at room temperature for 24 h to facilitate the formation of a stable hydrogel. Finally, the hydrogel was freeze-dried to obtain TpBpy/CS aerogel. The synthesis method of Tp/CS aerogel was similar to that of TpBpy/CS aerogel, with the exception that TpBpy was omitted.

### Batch experiments

Palladium chloride was dissolved in a hydrochloric acid solution at a specific concentration to prepare the Pd(ii) standard solutions. Batch adsorption experiments were used to study its adsorption performance. All adsorption experiments were performed at 25 °C with three parallel samples. Firstly, the adsorption capacity of TpBpy/CS aerogel for Pd(ii) at various pH levels (1–5) was studied. The initial concentration of Pd(ii) was maintained at 250 mg L^−1^. The HNO_3_ and NaOH solutions were used to adjust the pH of the solution. To explore the adsorption kinetic, 5 mg TpBpy/CS aerogel was added to 10.0 mL Pd(ii) solution with a concentration of 50 mg L^−1^. The concentration of Pd(ii) was monitored at the predetermined time intervals. For the adsorption isotherm experiments, 5 mg of TpBpy/CS aerogel were mixed with 10.0 mL solutions containing varying initial concentrations of Pd(ii) (ranging from 10 to 250 mg L^−1^) in polyethylene bottles. The mixture was oscillated for 20 h. Then the concentration of Pd(ii) was determined by ICP-OES. To investigate the selectivity of TpBpy/CS aerogel for Pd(ii) adsorption, multiple coexisting cations including Mg^2+^, Cu^2+^, Al^3+^, K^+^, Ni^2+^, Ca^2+^, Zn^2+^, Fe^3+^, and Na^+^ were introduced into the initial solution. The samples were soaked in the solution for 24 h, and the adsorption capacity of Pd(ii) was determined.

### Regeneration and recycling

To evaluate the reusability of the material, a solution containing 0.5 mol L^−1^ thiourea and 0.5 mol L^−1^ HNO_3_ was used as an elution to treat the Pd(ii)-loaded TpBpy/CS aerogel. A 5 mg TpBpy/CS aerogel was introduced into a 10.0 mL solution of 20 mg L^−1^ Pd(ii), where it underwent shaking for 24 h for adsorption at pH = 1. Subsequently, the Pd(ii)-loaded TpBpy/CS aerogel was eluted using a 5.0 mL mixture of 0.5 mol L^−1^ thiourea and 0.5 mol L^−1^ HNO_3_ solution, with shaking for 4 h. Prior to the next adsorption and desorption experiment, the TpBpy/CS aerogel was washed with ultrapure water until it reached a neutral pH. The reusability of the material was evaluated by observing the adsorption and desorption rates observed over multiple cycles of adsorption and desorption experiments.

## Data availability

All data can be found in the main text and ESI.†

## Author contributions

Yang Liu: investigation, software, writing – original draft. Weikang Guo: formal analysis, writing – review & editing. Jiale Liu: writing – review & editing. Haijuan Tao: writing – review & editing. Juan Yang: writing – review & editing. Qin Shuai: funding acquisition. Yusuke Yamauchi: writing – review & editing. Brian Yuliarto: writing – review & editing. Yusuke Asakura: writing – review & editing. Lijin Huang: conceptualization, writing – review & editing.

## Conflicts of interest

There are no conflicts to declare.

## Supplementary Material

SC-OLF-D4SC08674K-s001
