# Supplementary material for: Bipyridine covalent organic framework aerogel for highly selective recovery of palladium in wastewater
Source: Chem Sci. 2025 Mar 4;16(13):5745–54. doi: 10.1039/d4sc08674k (PMC11878236; doi:10.1039/d4sc08674k)
Supplement: SC-016-D4SC08674K-s001 [file SC-016-D4SC08674K-s001.pdf]

Supplementary Information (SI) for Chemical Science.
This journal is © The Royal Society of Chemistry 2025

Supporting Information

Bipyridine covalent organic framework aerogel for highly selective recovery of palladium in wastewater

Yang Liu [a], Weikang Guo [a], Jiale Liu [a], Hai-juan Tao [a], Juan Yang [b], Qin Shuai [a], Yusuke Yamauchi[c,d,e], Brian Yuliarto[f], Yusuke Asakura*[c], Lijin Huang*[a]

a. State Key Laboratory of Biogeology and Environmental Geology, Faculty of Materi-als Science and Chemistry, China University of Geosciences, No. 388, Lumo Road, Hongshan District, Wuhan 430074, PR China.

b. School of Chemistry and Environmental Engineering, Wuhan Institute of Technology, LiuFang Campus, No.206, Guanggu 1st Road, Donghu New & High Technology Development Zone, Wuhan, Hubei Province, Wuhan 430205, PR China

c. Department of Materials Process Engineering, Graduate School of Engineering, Nagoya University, Furo-cho, Chikusa-ku, Nagoya, Aichi 464-8603, Japan

d. Australian Institute for Bioengineering and Nanotechnology (AIBN), The University of Queensland, Brisbane, QLD 4072, Australia

e. Department of Chemical and Biomolecular Engineering, Yonsei University, 50 Yon-sei-ro, Seodaemun-gu, Seoul, 03722 South Korea

f. Advanced Functional Materials Laboratory, Department of Engineering Physics, Institute of Technology Bandung, Bandung 40132, Indonesia

1. Point of zero charge studies (pH_{PZC})

The point of zero charge of TpBpy/CS aerogel was determined by the solid addition method as reported earlier.[1] A series of solutions with initial pH (pH_i) 2.0 to 8.0 were prepared by adding HCl and NaOH to the 5.0 mL of 0.1 mol L^{-1} NaCl. Next, 5 mg of TpBpy/CS aerogel was added to each solution and left stirring for 24 hours (h) at room temperature. After 24 hours, the final pH (pH_f) of the solutions was measured and the plot of a difference between pH_f and pH_i was obtained ($\Delta pH=pH_f-pH_i$). The resultant plot derives a point of intersection which shows the point of zero charge for the TpBpy/CS aerogel.

2. Adsorption experiments

Batch adsorption experiments were performed at 25°C.

Removal efficiency (R) of Pd(II) and adsorption capacity Q_e (mg g^{-1}) at equilibrium were calculated by the following equations:

$$R = (C_0 - C_e) \times 100\% \quad (1)$$

$$Q_e = \frac{(C_0 - C_e)}{m} \times V \quad (2)$$

Where C_0 (mg L^{-1}) and C_e (mg L^{-1}) are the initial and equilibrium Pd(II) ion concentrations in the solution, respectively. Q_e (mg g^{-1}) is the equilibrium adsorption capacity, and V is the volume of solution (mL).

3. Adsorption Kinetics

To better understand the adsorption behavior of TpBpy/CS aerogel on Pd, pseudo 1st order and pseudo 2nd order models were used to analyze the experimental data.

The pseudo 1st order model: $Q_t = Q_e(1 - \exp(- k_1 \cdot t))$ (3)

The pseudo 2nd order model: $Q_t = \frac{k_2 Q_e^2 t}{1 + k_2 Q_e t}$ (4)

Where Q_t is adsorption capacity at time (mg g^{-1}), Q_e (mg·g^{-1}) represents the equilibrium adsorption capacity, k_1 (min^{-1}) and k_2 (g mg^{-1} min^{-1}) are rate constants of the pseudo 1st order and pseudo 2nd order kinetics, respectively.

4. Adsorption isotherms

The Langmuir adsorption model is given by: $Q_e = \frac{C_e Q_m K_L}{1 + K_L C_e}$ (5)

Where c_e and Q_e are the Pd solution concentration (mg L^{-1}) and equilibrium adsorption capacity (mg g^{-1}) when equilibrium is reached at a certain concentration, respectively. Q_m is the maximum adsorption capacity (mg g^{-1}), and K_L is the Langmuir adsorption rate constant.

The Freundlich adsorption model is expressed as follows: $Q_e = K_F C_e^{\frac{1}{n}}$ (6)

Where K_F and 1/n are the Freundlich adsorption constants. The parameter 1/n indicates the strength of adsorption. When 0.1< 1/n < 0.5, the adsorption process is favorable; when 0.5< 1/n < 1, the adsorption process is hindered; and when 1/n > 1, the adsorption rarely occurs.

5. Selectivity experiments

The Distribution coefficient (K_d) values were calculated by the following equations:

$$K_d = \frac{Q_e}{C_e} \quad (7)$$

The separation factors (*SF*) were calculated by the following equations:

$$SF = \frac{K_d(\mathrm{Pd})}{K_d(\text{other ions})} \quad (8)$$

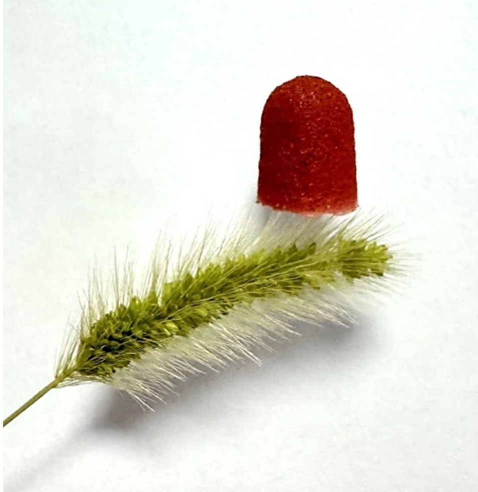

Fig. S1. The actual monolith of the TpBpy/CS aerogel.

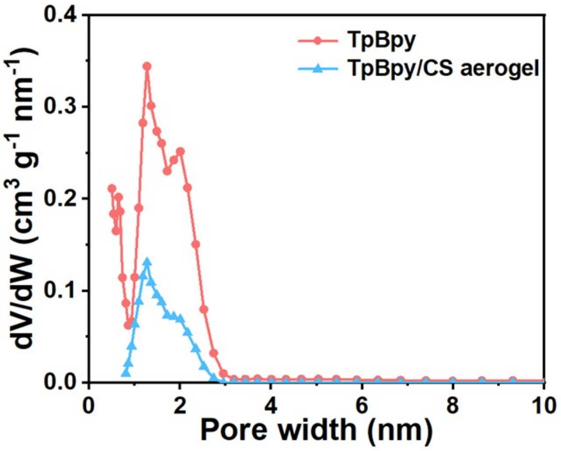

Fig. S2. Pore size distribution of TpBpy/CS aerogel and TpBpy.

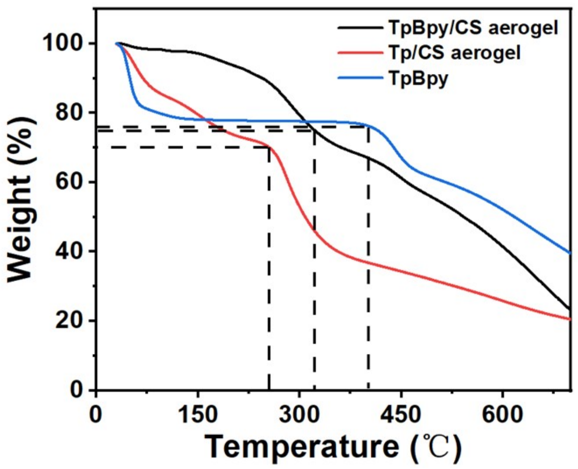

Fig. S3. TGA curves of TpBpy, Tp/CS aerogel and TpBpy/CS aerogel.

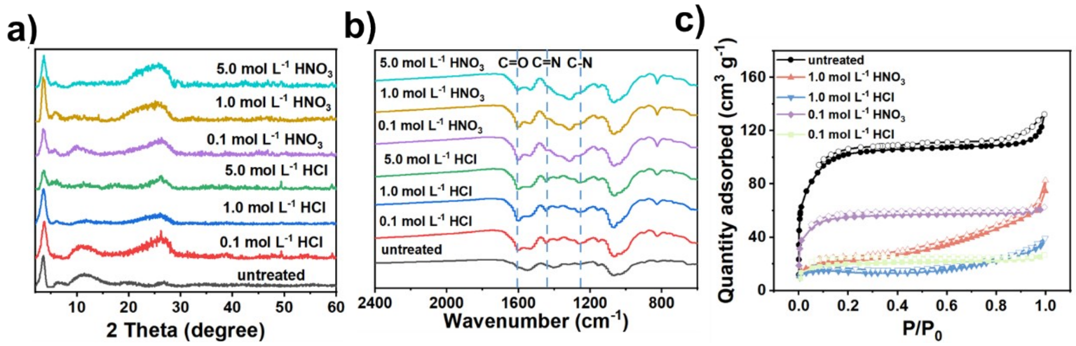

Fig. S4. a) XRD patterns, b) FT-IR spectra and c) N_2 adsorption-desorption isotherm curves of TpBpy/CS aerogels after soaked in 0.1, 1.0 and 5.0 mol L^{-1} HCl and HNO_3 solution for 24 h.

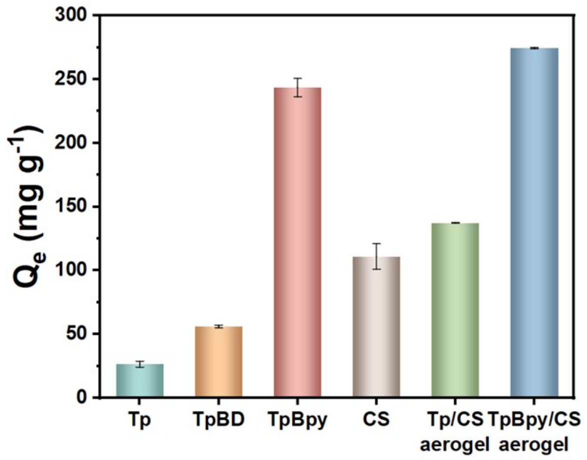

Fig. S5. Comparison of adsorption capacity of Tp, TpBD, CS, Tp/CS aerogel, TpBpy and TpBpy/CS aerogel for Pd(II) (m=5 mg, V=10.0 mL, C_{Pd}=250 mg L^{-1}, Temperature=25°C, pH=1, Time=24 h).

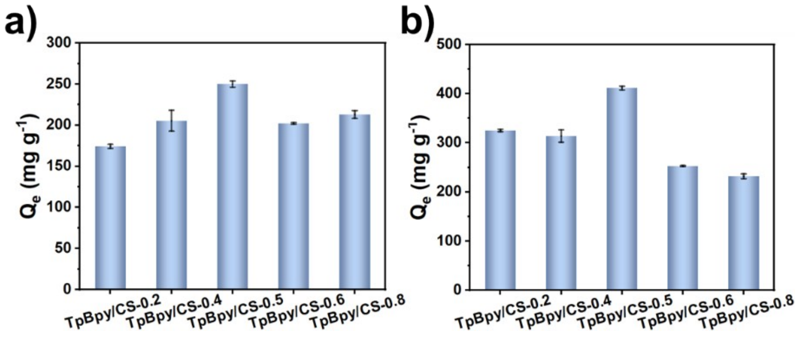

Fig. S6. a) Adsorption capacities of TpBpy/CS-*X* aerogel with different COF contents to Pd(II); b) adsorption capacities of COF to Pd(II) in TpBpy/CS-*X* aerogel with different COF contents, where the adsorption capacity is normalized based on the quantity of COF present in COF/CS aerogels (m=5 mg, V=10.0 mL, C_{Pd}=250 mg L^{-1}, Temperature =25°C, pH=1, Time=24 h).

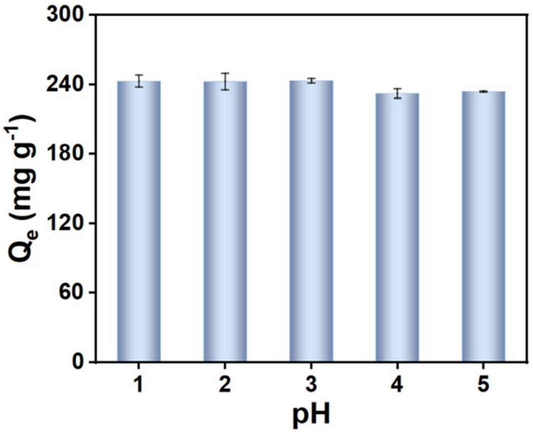

Fig. S7. Adsorption capacity of TpBpy for Pd(II) at pH 1-5 (m=5 mg, V=10.0 mL, C_{Pd}=250 mg L^{-1}, Temperature =25°C, Time=24 h).

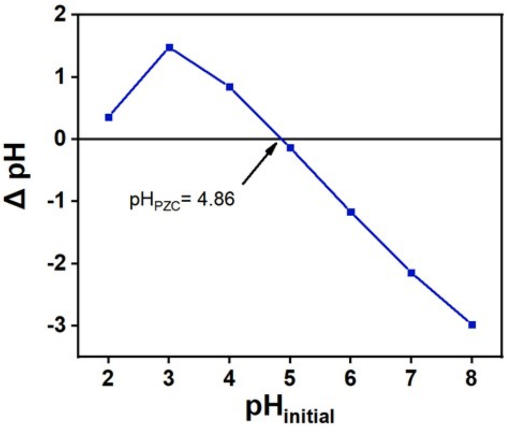

Fig. S8. pH_{PZC} of TpBpy/CS aerogel.

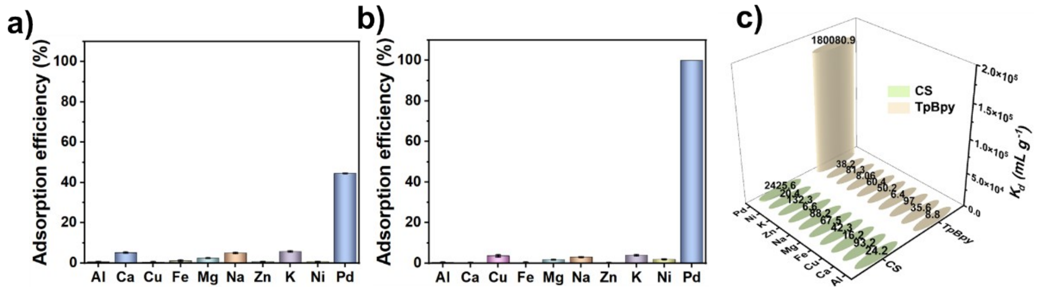

Fig. S9. The selectivity of a) CS and b) TpBpy, c) distribution coefficient (K_d) values of CS and TpBpy aerogel towards each element in the presence of coexisting ions (m=5 mg, V=10.0 mL, C_{Pd}=10 mg L^{-1}, C_{other}=1000 mg L^{-1}, Temperature =25°C, pH=1, Time=24 h).

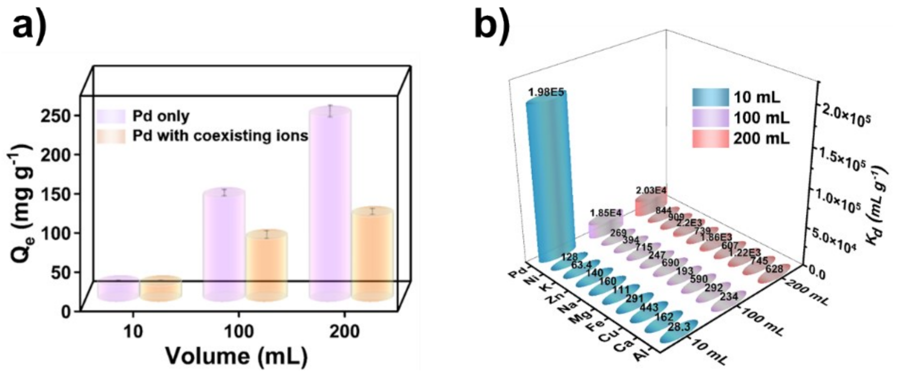

Fig. S10. a) Adsorption efficiency and adsorption capacity of Pd(II) by TpBpy/CS aerogel at different volumes (V=10, 100, or 200 mL, m=5 mg, C_{Pd}=10 mg L^{-1}, pH=1, T=25℃, t=24 h); b) Distribution coefficient (K_d) values of TpBpy/CS aerogel towards each element in the presence of coexisting ions at different volumes (V=10, 100, or 200 mL, m=5 mg, C_{Pd}=10 mg L^{-1}, C_{other}=1000 mg L^{-1}, pH=1, Temperature =25℃, Time=24 h).

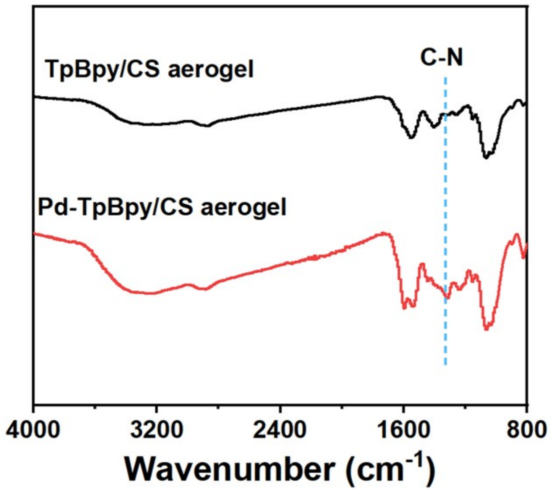

Fig. S11. FT-IR spectra of TpBpy/CS aerogel adsorption before and after Pd(II).

Table S1. The ingredients for the preparation of TpBpy/CS-*X* aerogels.

Aerogel	TpBpy (mg)	CS (mg)	Tp (mg)	Volume of water (μL)	Volume of AcOH (μL)	TpBpy/CS aerogel (mg)	The proportion of COF
TpBpy/CS-0.2	4.0	16	2.0	400	600	21	0.19
TpBpy/CS-0.4	8.0	12	2.0	400	600	21	0.38
TpBpy/CS-0.5	10	10	2.0	400	600	20	0.50
TpBpy/CS-0.6	12	8.0	2.0	400	600	21	0.57
TpBpy/CS-0.8	16	4.0	2.0	400	600	20	0.80

The *X* represents the weight% of COF in the TpBpy/CS-*X* aerogel ($X = \frac{m(\text{TpBpy})}{m(\text{TpBpy/CS aerogel})} \times 100\%$).

Table S2. Kinetic model parameters of Pd(II) adsorption by TpBpy/CS aerogel.

Adsorbent	Pseudo 1st order			Pseudo 2nd order		
	Q_e (mg g^{-1})	k_1 (min^{-1})	R^2	Q_e (mg g^{-1})	k_2 (g min^{-1} mg^{-1})	R^2
TpBpy/CS aerogel	96.1	0.049	0.991	104.4	0.0007	0.996

Table S3. Adsorption isotherm parameters of Pd(II) adsorption by TpBpy/CS aerogel.

Langmuir			Freundlich		
Q_m (mg g^{-1})	K_L	R^2	n	K_F	R^2
247.4	0.385	0.904	3.98	83.5	0.972

Table S4. The comparison for Pd(II) adsorption properties of the TpBpy/CS aerogel with other reported adsorbents.

Materials	Acidity of the solution	Adsorption equilibrium time (min)	Adsorption capacity (mg g^{-1})	Concentration of Pd in selectivity experiments (mg L^{-1})	The adsorption efficiency of Pd in the selective experiment (%)	Ref.
COP-1-Cl	pH=3.0	3	246.7	100	94.3	[2]
Tp-DG_{Cl}	pH=2.0	180	342.1	49.5	94.1	[3]
VP-AMPS-CS5	pH=3.0	360	184.9	50	49.3	[4]
TFBBpy-OMe-COF	pH=7.0	30	532.0	50	99.0	[5]
COF-316-DM	4.0 mol L^{-1} HNO_3	60	127.4	86.4	92.3	[6]
ECUT-COF-34	3.0 mol L^{-1} HCl	240	112.0	50	/	[7]
Tp-Azo-COF/SiO_2	3.0 mol L^{-1} HNO_3	120	85.4	/	/	[8]
CDCR	pH=3.0	150	204.3	100	99.9	[9]
DAVFs-CS	pH=3.0	660	207.0	100	20.0	[10]
TpBpy/CS aerogel	pH=1.0	120	274.4	10	99.9	This work

COP-1-Cl: covalent organic polymer-1-Cl.

Tp-DG_{Cl}: Tp is the abbreviation of 1,3,5-triformylphloroglucinol; DG is the abbreviation of 1,3-diaminoguanidine hydrochloride.

VP-AMPS-CS5: VP is the abbreviation of N-vinyl-2-pyrrolidone; AMPS is the abbreviation of 2-

acrylamido-2-methylpropane sulfonic acid; CS is the abbreviation of chitosan.

TFBBpy-OMe-COF: methoxy-containing covalent organic framework.

COF-316-DM: thiocarbamoyl-functionalized covalent organic framework.

ECUT-COF-34: East China University of Technology covalent organic framework-34.

Tp-Azo-COF-SiO_2: Tp is the abbreviation of 1,3,5-triformylphloroglucinol; Azo is the abbreviation of 4,4-Azodianiline.

CDCR: chitosan resin microsphere.

DAVFs-CS: dialdehyde viscose fibers- chitosan.

Table S5. The comparison for Pd(II) adsorption properties of the TpBpy/CS aerogel with other reported adsorbents at low concentrations.

Materials	Acidity of the solution	Dosage (g L^{-1})	Concentration of Pd (mg L^{-1})	The adsorption efficiency of Pd (%)	Adsorption capacity (mg g^{-1})	Ref.
Lyophilized algal cells	6.0 mol L^{-1} aqua regia	10.0	10.0	99.6	9.9	11
S-CTS	pH=2.5	0.8	1.0	99.7	1.2	12
SA-rGO	pH=11.0	1.0	0.1	99.0	0.085	13
MnHCF	pH=4.0	0.5	10.0	99.9	2.0	14
KTS-3	1.0 mol L^{-1} HNO_3	1.0	1.0	99.9	1.0	15
PAF-1-N_4	/	0.1	5.4	99.2	54.0	16
Meso-adsorbent	pH=3.5	0.5	5.0	99.0	9.9	17
TpBpy/CS aerogel	pH=1.0	0.5	10.0	99.9	20.0	This work

S-CTS: sulphur-containing chitosan.

SA-rGO: sulfamic acid reduced graphene oxide.

MnHCF: manganese hexacyanoferrate.

KTS-3: $K_{2x}Sn_{4-x}S_{8-x}$.

PAF-1-N: PAF is the abbreviation of porous aromatic framework.

Meso-adsorbent: mesoporous adsorbent.

Table S6. The $SF_{Pd/M}$ of CS, powdery TpBpy, and TpBpy/CS aerogel towards Pd and each element in solutions of varying volumes.

Metal ions	$SF_{Pd/M}$ (in simulated wastewater)					$SF_{Pd/M}$ (in actual wastewater)
	CS	TpBpy	TpBpy/CS aerogel			TpBpy/CS aerogel
			10 mL	100 mL	200 mL	
Ni	118.9	4714.1	1545.7	68.7	24.1	-
K	18.3	2215.1	3121.5	46.9	22.3	981.2
Zn	367.5	22342.5	1417.1	25.8	9.2	-
Na	27.5	2981.5	1233.4	74.9	27.5	-
Mg	35.9	3587.2	1776.9	26.7	10.9	498.5
Fe	57.3	2813.8	681.4	95.7	33.5	1027.6
Cu	149.7	1856.5	447.3	31.3	16.7	57.6
Ca	26.1	5058.4	1225.1	63.4	27.3	-
Al	100.2	20463.7	6991.5	78.9	32.4	-

Table S7. The composition of the real metallurgical wastewater from Jinbaoshan, Yunnan province, China, and the desorption solution after TpBpy/CS aerogel adsorption-desorption experiments.

Metal ions	Concentration (mg L^{-1})	
	The original solution	The desorption solution
Al	18.5	/
Na	322.5	/
Ni	76.3	/
Zn	3.7	/
Ca	5.3	/
K	6.3	0.2
Fe	18.1	0.7
Mg	1.4	0.04
Cu	33.1	10.9
Pd	19.2	38.3

Table S8. Selectivity of the TpBpy/CS aerogel in real wastewater.

Metal ions	K_d (mL g^{-1})
Fe	36
K	38
Mg	74
Cu	640
Pd	36892

Table S9. Prices for the calculation.

Materials	Packaging	Cost (RMB)
2,2'-bipyridine-5,5'-diamine	1 g	764
1, 3, 5-triformylphloroglucinol	1 g	117
chitosan	1 g	2
p-toluenesulfonic acid	1 g	3
acetic acid	500 mL	12
acetone	500 mL	23
N, N-dimethylacetamide	500 mL	50

Note: 7 RMB≈1 $.

References

1. V. Gomase, P. Doondani, D. Saravanan, S. Pandey and R. Jugade, A novel chitosan-barbituric acid hydrogel supersorbent for sequestration of chromium and cyanide ions: equilibrium studies and optimization through RSM, *Separation and Purification Technology*, 2024, **330**, 125475.
2. P. Wu, Y. Jiang, W. Lin, K. Wang, K. Wang, S. Qin, Y. Cai, L. Yuan and W. Feng, Novel cationic porous materials with open architectures for highly efficient palladium recovery: integrated experimental studies and DFT simulations, *Hydrometallurgy*, 2023, **221**, 106138.
3. L. Zhao, X. Ma, J. Xiong, Q. Zhou, W. Chen, Z. Yang, F. Jiang, S. Wang, X. Yang and H. Bai, Guanidinium-functionalized ionic covalent organic framework for selective and efficient recovery of palladium(II) from metallurgical wastewater, *Journal of Environmental Chemical Engineering*, 2023, **11**, 110549.
4. A. Haleem, F. Wu, M. Ullah, T. Saeed, H. Li and J. Pan, Chitosan functionalization with vinyl monomers via ultraviolet illumination under cryogenic conditions for efficient palladium recovery from waste electronic materials, *Separation and Purification Technology*, 2024, **329**, 125213.
5. Y. Zhao, C. Xu, Q. Qi, J. Qiu, Z. Li, H. Wang and J. Wang, Tailoring delicate pore environment of 2D covalent organic frameworks for selective palladium recovery, *Chemical Engineering Journal*, 2022, **446**, 136823.
6. F. Zhao, Y. Bai, X. Zhou, L. He, Y. Tao, J. Chen, M. Zhang, Q. Guo, Z. Ma, L. Chen, L. Zhu, T. Duan, Z. Chai and S. Wang, An aryl-ether-linked covalent organic framework modified with thioamide groups for selective extraction of palladium from strong acid solutions, *Chemistry - A European Journal*, 2023, **29**, e202302445.
7. L. Zhong, Z. Yu, Q. Zhang, X. Feng and F. Luo, Construction of multiple-component covalent organic frameworks by an in-situ approach for boosting palladium recovery from strong acid, *ACS Materials Letters*, 2023, **5**, 2665-2672.
8. S. Xu, S. Ning, X. Wang, F. Gao, L. Chen, X. Yin, T. Fujita and Y. Wei, Silica-based covalent organic framework composite for efficient separation and enrichment of palladium and its heterogeneous catalysis application, *Separation and Purification Technology*, 2023, **327**, 124977.
9. L. Chen, K. Wu, M. Zhang, N. Liu, C. Li, J. Qin, Q. Zhao and Z. Ye, Synthesis of carbon disulfide modified chitosan resin and its adsorption properties for palladium(II) in wastewater, *Chemical Engineering Journal*, 2023, **466**, 143082.
10. F. Liu, S. Hua, L. Zhou and B. Hu, Development and characterization of chitosan functionalized dialdehyde viscose fiber for adsorption of Au(III) and Pd(II), *International Journal of Biological Macromolecules*, 2021, **173**, 457-466.
11. A. Minoda, S. I. Miyashita, T. Kondo, T. Ogura, J. Sun and Y. Takahashi, Low-concentration palladium recovery from diluted aqua regia-based wastewater using lyophilized algal cells, *Resources, Conservation & Recycling Advances*, 2023, **17**, 200140.
12. Q. Xie, T. Lin, F. Chen, D. Wang and B. Yang, Recovery of ultra-trace palladium using chitosan and its sulphur-containing derivative in HCl medium, *Hydrometallurgy*, 2018, **178**, 188-194.
13. L. Chen, Y. L. Li, R. X. Li, Q. J. Shu and T. Yang, Three-dimensional self-assembled

reduced graphene oxide composite as a promising adsorbent for the collection of palladium (II) and platinum (IV) in a low concentration, *Journal of the Iranian Chemical Society*, 2023, **20**, 2861-2870.
14. M. Liu, Y. Du, Y. Liu, X. Li, S. Yang, J. Feng, Z. Huang, Y. Chen, B. Wang and R. Chen, Rapid separation of the low concentration Pd from Pd-Pt coexisting systems: Cyano-group's monomer-specific affinity, *Journal of Colloid and Interface Science*, 2024, **665**, 422-429.
15. A. D. Pournara, J. H. Tang, L. Yang, J. T. Liu, X. Y. Huang, M. L. Feng and M. G. Kanatzidis, Leveraging hard-soft acid-base interactions for effective palladium capture in acidic solutions, *Chemistry of Materials*, 2024, **36**, 3013-3021.
16. T. Ma, Z. Li, R. Zhao, J. Song, Y. Tian, S. Ding and G. Zhu, Nanoporous aromatic framework with bulky binding groups for palladium recovery, *ACS Applied Nano Materials*, 2022, **5**, 15115-15122.
17. M. R. Awual, M. A. Khaleque, Y. Ratna and H. Znad, Simultaneous ultra-trace palladium(II) detection and recovery from wastewater using new class meso-adsorbent, *Journal of Industrial and Engineering Chemistry*, 2015, **21**, 405-413.
